# Correction: Modification of silica nanoparticles by 2,4-dihydroxybenzaldehyde and 5-bromosalicylaldehyde as new nanocomposites for efficient removal and preconcentration of Cu(ii) and Cd(ii) ions from water, blood, and fish muscles

**DOI:** 10.1039/d2ra90073d

**Published:** 2022-07-19

**Authors:** Hanem M. Gad, S. M. El Rayes, Ehab A. Abdelrahman

**Affiliations:** Chemistry Department, Faculty of Science, Suez Canal University Ismailia 41522 Egypt; Chemistry Department, Faculty of Science, Benha University Benha 13518 Egypt dr.ehabsaleh@yahoo.com ehab.abdelrahman@fsc.bu.edu.eg +201010636875

## Abstract

Correction for ‘Modification of silica nanoparticles by 2,4-dihydroxybenzaldehyde and 5-bromosalicylaldehyde as new nanocomposites for efficient removal and preconcentration of Cu(ii) and Cd(ii) ions from water, blood, and fish muscles' by Hanem M. Gad *et al.*, *RSC Adv.*, 2022, **12**, 19209–19224, https://doi.org/10.1039/D2RA03177A.

The authors regret that the units of the concentration in Tables 7, 8, 9, 10, 11 and 12 were not correctly given in the original article. In Tables 7 and 8, the tolerance limit should be given in μg L^−1^. In Tables 9, 10, 11, and 12 the found concentration should be given in μg L^−1^. The corrected versions of the tables are shown below.

**Table:** Removal of Cu(ii) and Cd(ii) ions from binary mixtures using N_1_ nanocomposite in the presence of different diverse ions

Diverse ion	Tolerance limit (μg L^−1^)	% *R*	
		Cu(ii)	Cd(ii)
K(i)	900	99.15	99.96
Na(i)	900	99.24	99.65
Ca(ii)	120	98.38	99.08
Ba(ii)	80	99.08	99.18
Mg(ii)	120	97.17	99.00
Hg(ii)	80	98.57	98.37
Fe(ii)	120	97.39	99.14
Mn(ii)	80	96.79	97.76
Ni(ii)	80	95.87	96.79
Al(iii)	80	98.16	98.45
Fe(iii)	100	99.46	99.53
HCO_3_^−^	1000	99.68	99.78
NO_3_^−^	1000	99.73	99.65
Cl^−^	1000	99.82	99.72
SO_4_^2−^	1000	99.18	99.64

**Table:** Removal of Cu(ii) and Cd(ii) ions from binary mixtures using N_2_ nanocomposite in the presence of different diverse ions

Diverse ion	Tolerance limit (μg L^−1^)	% *R*	
		Cu(ii)	Cd(ii)
K(i)	900	99.26	99.78
Na(i)	900	99.37	99.89
Ca(ii)	120	98.26	99.16
Ba(ii)	80	99.65	99.32
Mg(ii)	120	97.28	99.43
Hg(ii)	80	98.54	98.52
Fe(ii)	120	97.31	98.95
Mn(ii)	80	96.64	98.15
Ni(ii)	80	95.77	96.64
Al(iii)	80	98.53	98.83
Fe(iii)	100	99.36	99.73
HCO_3_^−^	1000	99.47	99.58
NO_3_^−^	1000	99.17	99.28
Cl^−^	1000	99.63	99.71
SO_4_^2−^	1000	99.13	99.82

**Table:** Determination of Cu(ii) ions in real samples using N_1_ nanocomposite

Sample	Added volume from Cu(ii) stock solution (1000 μg L^−1^)								
	0 mL			0.2 mL			0.4 mL		
	Found concentration (μg L^−1^)	% Recovery	% RSD	Found concentration (μg L^−1^)	% Recovery	% RSD	Found concentration (μg L^−1^)	% Recovery	% RSD
Sea water	4.600 ± 0.124	—	2.174	7.040 ± 0.142	97.139	1.619	9.62 ± 0.309	97.362	2.589
Tap water	8.320 ± 0.492	—	2.150	10.816 ± 0.255	98.709	1.901	13.580 ± 0.492	99.993	2.918
Fish muscles	7.230 ± 0.247	—	2.749	9.820 ± 0.254	99.490	2.087	12.400 ± 0.351	99.226	2.281
Blood	1.090 ± 0.028	—	2.051	3.580 ± 0.104	95.551	2.337	6.358 ± 0.053	99.511	0.671

**Table:** Determination of Cu(ii) ions in real samples using N_2_ nanocomposite

Sample	Added volume from Cu(ii) stock solution (1000 μg L^−1^)								
	0 mL			0.2 mL			0.4 mL		
	Found concentration (μg L^−1^)	% Recovery	% RSD	Found concentration (μg L^−1^)	% Recovery	% RSD	Found concentration (μg L^−1^)	% Recovery	% RSD
Sea water	4.500 ± 0.124	—	2.222	6.990 ± 0.188	97.795	2.169	9.540 ± 0.272	97.534	2.297
Tap water	8.300 ± 0.152	—	1.476	10.876 ± 0.141	99.438	1.043	13.500 ± 0.412	99.550	2.457
Fish muscles	7.060 ± 0.225	—	2.573	9.660 ± 0.299	99.579	2.493	12.260 ± 0.208	99.451	1.365
Blood	1.082 ± 0.025	—	1.894	3.600 ± 0.088	96.290	1.964	6.280 ± 0.195	98.413	2.495

**Table:** Determination of Cd(ii) ions in real samples using N_1_ nanocomposite

Sample	Added volume from Cd(ii) stock solution (1000 μg L^−1^)								
	0 mL			0.2 mL			0.4 mL		
	Found concentration (μg L^−1^)	% Recovery	% RSD	Found concentration (μg L^−1^)	% Recovery	% RSD	Found concentration (μg L^−1^)	% Recovery	% RSD
Sea water	0.184 ± 0.007	—	2.977	2.720 ± 0.104	95.671	3.076	5.390 ± 0.092	98.213	1.376
Tap water	0.146 ± 0.005	—	2.837	2.754 ± 0.063	98.168	1.844	5.372 ± 0.103	98.560	1.549
Fish muscles	21.100 ± 0.679	—	2.596	23.400 ± 0.519	98.719	1.788	26.200 ± 0.555	99.646	1.707
Blood	1.068 ± 0.037	—	2.762	3.564 ± 0.119	95.685	2.696	6.348 ± 0.132	99.696	1.672

**Table:** Determination of Cd(ii) ions in real samples using N_2_ nanocomposite

Sample	Added volume from Cd(ii) stock solution (1000 μg L^−1^)								
	0 mL			0.2 mL			0.4 mL		
	Found concentration (μg L^−1^)	% Recovery	% RSD	Found concentration (μg L^−1^)	% Recovery	% RSD	Found concentration (μg L^−1^)	% Recovery	% RSD
Sea water	0.171 ± 0.006	—	2.905	2.740 ± 0.068	96.802	1.999	5.450 ± 0.088	99.534	1.297
Tap water	0.157 ± 0.006	—	2.848	2.774 ± 0.054	98.503	1.563	5.440 ± 0.142	99.612	2.096
Fish muscles	21.600 ± 0.473	—	1.763	24.000 ± 0.760	99.165	2.552	26.730 ± 0.890	99.774	2.683
Blood	1.078 ± 0.038	—	2.814	3.594 ± 0.089	96.232	2.002	6.334 ± 0.137	99.321	1.742

The Royal Society of Chemistry apologises for these errors and any consequent inconvenience to authors and readers.

## Supplementary Material

